# Trends in the epidemiology of larynx and lung cancer in south-east England, 1985–2004

**DOI:** 10.1038/sj.bjc.6604787

**Published:** 2008-11-18

**Authors:** V H Coupland, P Chapman, K M Linklater, A Sehgal, H Møller, E A Davies

**Affiliations:** 1 King's College London, Thames Cancer Registry, 42 Weston Street, London, SE13QD, UK; 2 Royal Surrey County Hospital, Egerton Road, Guildford, Surrey, GU2 5XX, UK

**Keywords:** larynx cancer, lung cancer, epidemiology, trends, incidence

## Abstract

We analysed data on 8987 larynx and 174060 lung cancer patients diagnosed between 1985 and 2004, of which 17.3% of larynx and 35.5% of lung cancers were in females. The age-standardised rates for each cancer declined in both sexes, but since the 1990s, the rates in females over 70 years of age have been diverging.

It is informative to compare trends in the epidemiology of larynx and lung cancer, as they share several characteristics. Both are more common among males, predominately affect older adults, and are strongly associated with tobacco smoking and socioeconomic deprivation (Thorne *et al*, 1997; Brewster and Møller, 2005; Wood *et al*, 2005; Groome *et al*, 2006; DAHNO, 2007; Quinn *et al*, 2001a, 2001b). In 1994, the majority of larynx cancer in England and Wales was recorded as occurring in the area of the glottis, followed by the supraglottis, with a small proportion in the subglottis and laryngeal cartilages. A large proportion remained unspecified (Quinn *et al*, 2001a).

Smoking, particularly of cigarettes, is a well-established risk factor for both larynx and lung cancer (IARC, 1986, 2002). Alcohol consumption is also independently associated with larynx cancer (IARC, 1988; Altieri *et al*, 2002; Talamini *et al*, 2002; Bosetti *et al*, 2002a; Vaezi *et al*, 2006), and the joint effect of smoking and alcohol is approximately multiplicative (Tuyns *et al*, 1988; Talamini *et al*, 2002; Altieri *et al*, 2005). Alcohol consumption is not considered as a causal factor in lung cancer (IARC, 1988), although a weak increase in risk has been observed in one meta-analysis (Bagnardi *et al*, 2001).

Another risk factor for larynx cancer is gastro-oesophageal reflux disease (GORD), especially in heavy smokers (Vaezi *et al*, 2006). Poor diet, human papillomaviruses (HPVs), and exposure to asbestos and other occupational hazards have also been implicated, but the evidence is less conclusive (Cattaruzza *et al*, 1996; Quinn *et al*, 2001a; Bosetti *et al*, 2002b; Olshan, 2006). Although smoking is the dominant risk factor for lung cancer, occupational exposures to industrial carcinogens also increase the risk (Quinn *et al*, 2001b; Wood *et al*, 2005).

In males over the age of 45 years, the risk of developing larynx cancer is around five times higher than in females (DAHNO, 2007), and consequently epidemiological studies can rarely include large numbers of females. The Thames Cancer Registry covers an area of south-east England, which by 2004 included a population of 14 million residents. The size of its database provided an opportunity to compare trends in the incidence of larynx and lung cancer in both sexes between 1985 and 2004.

## Materials and methods

In the United Kingdom, cancer registries record the occurrence of new cases of cancer in their residential populations. During the study period, the Thames Cancer Registry covered a population living in Essex, Hertfordshire, London, Kent, Surrey, and Sussex. In this area, registration is initiated by pathology and clinical information received from hospitals. Information about death is provided by the National Health Service Central Register through the Office for National Statistics. Trained data collection officers seek further information from the medical records on demographic details, tumour characteristics including disease stage, and treatment. Data are added to a central database, quality-assured, and are updated continuously.

We extracted data on 8987 patients (7429 males and 1558 females) with larynx cancer (ICD-10 C32) and 174 060 patients (112 333 males and 61 727 females) with lung cancer (ICD-10 C33-C34) diagnosed between 1985 and 2004 and resident in the area. We aggregated data into the main morphology groups: adenocarcinoma, small cell carcinoma, squamous cell carcinoma, other, and unspecified. For larynx cancer, we considered the anatomical subgroups of glottis, supraglottis, laryngeal cartilage, and not otherwise specified. We calculated directly age-standardised incidence rates using the European standard population for the four periods: 1985–1989, 1990–1994, 1995–1999, and 2000–2004 for males and females separately. We then calculated the average annual percentage change in incidence per 5-year period of diagnosis using weighted log-linear regression. We also calculated truncated age-standardised rates for the age groups (in years): 0–49, 50–59, 60–69, 70–79, and 80 plus.

## Results

Table 1 shows the smaller proportion of larynx (17.3%) and lung cancer (35.5%) diagnoses in females. Most larynx cancers were recorded as squamous cell carcinoma (83.3% in males and 77.2% in females). This proportion was lower in lung cancer (27.8% in males and 19.1% in females) where more were adenocarcinoma, small cell carcinoma, and other specified morphologies. Approximately 47.1% of male and 50.2% of female lung cancer patients had unspecified morphology. In larynx cancer, the glottis and supraglottis were the two main sites making up over one-half of all tumours. The proportion of females with glottis cancer was lower (26.4%) than for males (42.4%), whereas cancer of the supraglottis was more common in females (24.4%) than males (14.2%). However, a large proportion of cancers in each sex had no information on anatomical subsite (42.1% of males and 45.1% of females).

**Figure 1:**
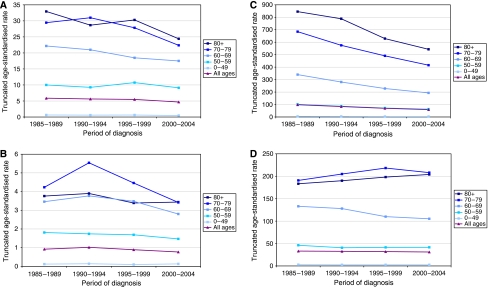
Trends in truncated age-standardised rates for larynx cancer and lung cancer in male and female residents of south-east England, 1985–2004. (**A**) Larynx cancer in males. (**B**) Larynx cancer in females. (**C**) Lung cancer in males. (**D**) Lung cancer in females.

Figure 1 shows the age-standardised and truncated age-standardised incidence rates for larynx and lung cancer. In both cancers, the rates were much lower in females than in males. The greatest decline over time was in male lung cancer, with the percentage change in incidence per 5-year period of diagnosis being −15.6% compared with only −2.1% in females. The rates for larynx cancer also declined in both sexes, with a percentage change per 5-year period of diagnosis of −6.6% in males and −6.3% in females. The truncated age-standardised incidence rates for both larynx and lung cancer in men were highest in the over-70 age groups, whereas in women, they were highest in the 70–79 age group. The rates of female larynx and lung cancer in the over-70 age group diverged from the early 1990s, with rates in larynx cancer declining and those in lung cancer increasing. In larynx cancer, rates in the female 70–79 age group declined faster than in the 80 plus group, whereas in lung cancer, the rate in the female 80 plus group increased faster than in the 70–79 age group. Age-standardised rates by birth cohort showed stronger trends in lung cancer compared with larynx cancer between 1900 and 1950 (data not shown). In male lung cancer, rates declined in successive generations and female rates increased, whereas in both sexes, larynx cancer rates appeared fairly stable across birth cohorts.

## Discussion

This study of the epidemiology of larynx and lung cancer in south-east England found that both cancers affected predominantly males aged over 70 years, and that females represented 17.3% of larynx and 35.5% of lung cancer diagnoses. The age-standardised incidence rates for each cancer declined in both sexes between 1985 and 2004, but to a greater extent for lung cancer in males. The incidence of larynx and lung cancer declined in males in all age groups over 60 years, but the incidence of these two cancers in females aged over 70 years diverged from the early 1990s. Whereas the incidence of lung cancer increased in the 70–79 and 80 plus age groups, the rates for larynx cancer declined in these groups.

The main limitation of our study is that it was based on cancer registration data, which relies on the information available in medical records. Data on disease morphology were incomplete, a large proportion of larynx cancer cases had no information on anatomical subsite, and information on individual risk factors such as smoking and alcohol consumption is not available. Although data from a 20-year period for approximately one-quarter of the English population provided sufficient numbers of female patients with larynx cancer to allow more detailed description of its epidemiology than previously, the incidence rates were still based on relatively small numbers.

Some findings of our study, such as the predominance of male patients, and the decline in the age-standardised incidence rates of both cancers up until 2004 are similar to those reported previously by national studies (Quinn *et al*, 2001a, 2001b; Brewster and Møller, 2005; Wood *et al*, 2005; DAHNO, 2007). The new finding of diverging trends for the two cancers in females over 70, with larynx cancer rates declining, whereas the lung cancer rates increase is surprising. This could reflect past differences in smoking behaviours between males and females, or possibly a decline in the effect of additional risk factors for larynx cancer, including alcohol consumption, GORD, poor diet, and HPV infection (Cattaruzza *et al*, 1996; Quinn *et al*, 2001a; Bosetti *et al*, 2002b; Olshan, 2006; Vaezi *et al*, 2006). A study of 33 European countries between 1980 and 2001 found stronger similarities in mortality trends between larynx and other alcohol-related cancers than between larynx and lung cancer, suggesting that alcohol consumption is most likely to be important (Bosetti *et al*, 2006).

The large proportion of larynx cancer assigned to unknown anatomical subsite emphasises the need for better recording of this information after surgery and its transfer to the cancer registry to enable comparative analyses across the regions and over time (Department of Health, 2007). Several findings of this study could be explored using larger data sets. First, our finding of a decline in the age-standardised rate of larynx cancer in males and females in south-east England should be compared with other regions of the United Kingdom and other countries. Second, investigation could compare the trends in different age groups between larynx and other alcohol-related cancers. Third, the higher proportion of supraglottis larynx cancer in females may be an artefact of incomplete data, but could be explored in other regions in case there may be a higher risk for female disease at this site. Finally, the decline in the incidence of both cancers over the 20 years is a positive finding. However, the continuing increase of lung cancer among older females emphasises the need to continue to develop smoking cessation programmes to reach this group, as there is good evidence that individuals who give up smoking in middle age can avoid the majority of their subsequent risk (Peto *et al*, 2000).

## Figures and Tables

**Table 1:** Clinical characteristics of patients diagnosed with larynx and lung cancer in south-east England, 1985–2004

	**Males**	**Females**
**Larynx cancer (ICD10 C32)**	***N* (%)**	***N* (%)**
All cases	7429 (82.7)	1558 (17.3)
		
*Morphology*		
Adenocarcinoma	27 (0.4)	18 (1.2)
Small cell carcinoma	11 (0.1)	12 (0.8)
Squamous cell carcinoma	6190 (83.3)	1202 (77.2)
Other specified morphologies	73 (1.0)	16 (1.0)
Unspecified morphologies	1128 (15.2)	310 (19.9)
		
*Anatomical subsite*		
Glottis	3149 (42.4)	411 (26.4)
Supraglottis	1054 (14.2)	380 (24.4)
Subglottis	76 (1.0)	55 (3.5)
Laryngeal cartilage	25 (0.3)	10 (0.6)
Larynx (not otherwise specified)	3125 (42.1)	702 (45.1)
		
**Lung cancer (ICD10 C33-C34)**		
All cases	112333 (64.5)	61727 (35.5)
		
*Morphology*		
Adenocarcinoma	11889 (10.6)	8861 (14.4)
Small cell carcinoma	11822 (10.5)	7702 (12.5)
Squamous cell carcinoma	31229 (27.8)	11765 (19.1)
Other specified morphologies	4479 (4.0)	2441 (4.0)
Unspecified morphologies	52914 (47.1)	30958 (50.2)

## References

[bib1] Altieri A, Bosetti C, Talamini R, Gallus S, Franceschi S, Levi F, Dal Maso L, Negri E, La Vecchia C (2002) Cessation of smoking and drinking and the risk of laryngeal cancer. Br J Cancer 87: 1227–1229 12439710 10.1038/sj.bjc.6600638 PMC2408914

[bib2] Altieri A, Garavello W, Bosetti C, Gallus S, La Becchia C (2005) Alcohol consumption and risk of laryngeal cancer. Oral Oncol 41: 956–965 15927525 10.1016/j.oraloncology.2005.02.004

[bib3] Bagnardi V, Blangiardo M, La Vecchia C, Corrao G (2001) Alcohol consumption and the risk of cancer: A meta-analysis. Alcohol Res Health 25(4): 263–269 11910703 PMC6705703

[bib4] Bosetti C, Gallus S, Franceschi S, Levi F, Bertuzzi M, Negri E, Talamini R, La Vecchia C (2002a) Cancer of the larynx in non-smoking alcohol drinkers and in non-drinking tobacco smokers. Br J Cancer 87: 516–518 12189548 10.1038/sj.bjc.6600469 PMC2376150

[bib5] Bosetti C, Talamini R, Levi F, Negri E, Franceschi S, Airoldi L, La Vecchia C (2002b) Fried foods: a risk factor for laryngeal cancer? Br J Cancer 87: 1230–1233 12439711 10.1038/sj.bjc.6600639 PMC2408902

[bib6] Bosetti C, Garavello W, Levi F, Lucchini F, Negri E, LaVecchia C (2006) Trends in laryngeal cancer mortality in Europe. Int J Cancer 119: 673–681 16496411 10.1002/ijc.21855

[bib7] Brewster D, Møller H (2005) Larynx. In Cancer Atlas of the United Kingdom and Ireland 1991–2000 Quinn M, Wood H, Cooper N and Rowan S (eds), pp 111–118. Ashford Colour Press Ltd: UK

[bib8] Cattaruzza MS, Maisonneuve P, Boyle P (1996) Epidemiology of Laryngeal Cancer. Oral Oncol 32B(5): 293–305 10.1016/0964-1955(96)00002-4 8944832

[bib9] DAHNO (2007) Key findings from the National Head and Neck Cancer Audit: Key findings for England and Wales for the audit period October 2005 to November 2006. DAHNO Second Annual Report. Leeds: Health and Social Care Information Centre

[bib10] Department of Health (2007) Cancer Reform Strategy. Available (online) http://www.dh.gov.uk/en/Publicationsandstatistics/Publications/PublicationsPolicyAndGuidance/DH_081006 (accessed 2nd July 2008)

[bib11] Groome P, Schulze K, Keller S, Mackillop W, O'Sullivan B, Irish J, Bissett R, Dixon P, Eapen L, Gulavita S, Hammond J, Hodson D, Mackenzie R, Schneider K, Warde P (2006) Explaining socioeconomic status effects in laryngeal cancer. Clin Oncol 18: 283–292 10.1016/j.clon.2005.12.010 16703745

[bib12] IARC (1986) Monographs on the Evaluation of Carcinogenic Risks to Human, Vol 38: Tobacco Smoking. IARC: Lyon

[bib13] IARC (1988) Monographs on the Evaluation of Carcinogenic Risks to Human, Vol 44: Alcohol Drinking. IARC: Lyon PMC6421508 3236394

[bib14] IARC (2002) Monographs on the Evaluation of Carcinogenic Risks to Humans, Vol 83; Tobacco Smoke and Involuntary Smoking. IARC: Lyon PMC4781536 15285078

[bib15] Olshan A (2006) Cancer of the larynx. In Cancer Epidemiology and Prevention, Schottenfeld D, Fraumeni J (eds), 3rd ed, pp 627–637. Oxford University Press: New York

[bib16] Peto R, Darby S, Deo H, Silcocks P, Whitley E, Doll R (2000) Smoking, smoking cessation, and lung cancer in the UK since 1950: combination of national statistics with two case-control studies. Brit Med J 321: 323–329 10926586 10.1136/bmj.321.7257.323 PMC27446

[bib17] Quinn M, Babb P, Brock A, Kirby L, Jones J (2001a) Larynx in Cancer trends in England and Wales 1950–1999. Studies on Medical and Population Subjects No. 66. The Stationary Office: London

[bib18] Quinn M, Babb P, Brock A, Kirby L, Jones J (2001b) Lung in Cancer trends in England and Wales 1950–1999. Studies on Medical and Population Subjects No. 66. The Stationary Office: London

[bib19] Talamini R, Bosetti C, La Vecchia C, Dal Maso L, Levi F, Bidoli E, Negri E, Pasche C, Vaccarella S, Barzan L, Franceschi S (2002) Combined effect of tobacco and alcohol on laryngeal cancer risk: a case-control study. Cancer Causes Control 13: 957–964 12588092 10.1023/a:1021944123914

[bib20] Thorne P, Etherington D, Birchall M (1997) Head and neck cancer in the south west of England: influence of socio-economic status on incidence and second primary tumours. Eur J Surg Oncol 23: 503–508 9484919 10.1016/s0748-7983(97)92917-6

[bib21] Tuyns A, Esteve J, Raymond L, Berrino F, Benhamou E, Blanchet F, Boffetta P, Crosignani P, Del Moral A, Lehmann W, Merletti F, Pequignot G, Riboli E, Sancho-Garnier H, Terracini B, Zubiri A, Zubiri L (1988) Cancer of the larynx/hypopharynx, tobacco and alcohol: IARC International Case-Control Study in Turin and Varese (Italy), Zaragoza and Navarra (Spain), Geneva (Switzerland) and Calvados (France). Int J Cancer 41: 483–491 3356483 10.1002/ijc.2910410403

[bib22] Vaezi M, Qadeer M, Lopez R, Colabianchi N (2006) Laryngeal cancer and gastroesophageal reflux disease: a case–control study. Am J Med 119: 768–776 16945612 10.1016/j.amjmed.2006.01.019

[bib23] Wood H, Cooper N, Rowan S, Quinn M (2005) Lung. In Cancer Atlas of the United Kingdom and Ireland 1991–2000 Quinn M, Wood H, Cooper N and Rowan S (eds), pp 111–118. Ashford Colour Press Ltd: UK

